# Age and First Seizure Length in Electroconvulsive Therapy

**DOI:** 10.1001/jamanetworkopen.2025.12092

**Published:** 2025-05-22

**Authors:** Alexander Sartorius, Charles H. Kellner, Sebastian Karl, Randall T. Espinoza

**Affiliations:** 1 Department of Psychiatry and Psychotherapy, Central Institute of Mental Health, Medical Faculty Mannheim, University of Heidelberg, Mannheim, Germany; 2 Department of Psychiatry and Behavioral Sciences, Medical University of South Carolina, Charleston; 3 Department of Psychiatry and Biobehavioral Sciences, Geffen School of Medicine at the University of California, Los Angeles; 4 University of Washington School of Medicine, Seattle; 5 Garvey Institute Center for Neuromodulation, University of Washington Northwest–Center for Behavioral Health and Learning, Seattle

## Abstract

This cohort study evaluates the association between patient age and first seizure length in electroconvulsive therapy.

## Introduction

The seizure at the first electroconvulsive therapy (ECT) treatment session is a unique event. It is typically the longest lasting in the course, and its duration may be related to how it is elicited; for example, if the electrical stimulus is only slightly above seizure threshold (ST), the seizure is likely to be long. Conversely, stimuli that are very much above ST are likely to produce shorter seizures. This phenomenon is particularly true at subsequent ECT sessions and calls into question the nearly universal policy of increasing stimulus dose when a seizure is considered too short. In a very large dataset, Luccarelli et al^1^ demonstrated that higher stimulus doses, in fact, result in shorter seizures. Furthermore, Gillving et al^2^ recently reported a relationship between seizure duration and depression response using Swedish registry data. Based on electroencephalogram (EEG) seizure duration at the initial (first) treatment in right unilateral (RUL) ECT and patient self-ratings, they found that patients with longer initial seizures had higher remission rates.^2^ However, higher stimulus doses are also known to be associated with better antidepressant outcomes. As there is no information on the subsequent seizure durations in the Swedish registry, this paradoxical situation could be explained by the hypothesis that the first seizure is not only exceptional but also, paradoxically, longer in older patients than in younger ones.

## Methods

We tested our hypothesis by taking a published database of patients treated at the Central Institute of Mental Health in Mannheim, Germany, between January 2010 and March 2021.^3^ This cohort study followed Strengthening the Reporting of Observational Studies in Epidemiology (STROBE) reporting guidelines. The retrospective analysis was approved by the ethics committee of the medical faculty Mannheim of the University of Heidelberg. The need for informed consent was waived because data were deidentified. To test for EEG seizure duration depending on age and charge, we used a general linear regression model (GLM) for data from the first ECT session exclusively. Statistical analyses were performed using StataSE version 17 (StataCorp) at a 2-sided significance level of α = .05.

## Results

Our analysis included 351 patients with a mean (SD) age of 63.2 (19.5) years. RUL electrode placement was used in 279 patients (79.5%), the mean (SD) charge was 57.9% (21.5%) of 504 mC, and the mean (SD) EEG seizure duration was 57.6 (18.1) seconds. Results from the GLM showed that higher age was associated with longer seizure duration (age: *z* = 2.08; coefficient for age, 0.28; 95% CI, 0.02-0.55; *P* = .04) (Figure). Charge and the interaction of age and charge showed no significant association. These results remained robust when excluding patients receiving propofol or thiopental as anesthetic medication (268 patients; age: *z* = 2.51; coefficient for age, 0.34; 95% CI, 0.07-0.60; *P* = .01) or when excluding patients treated with bilateral electrode placement (279 patients; age: *z* = 2.99; coefficient for age, 0.38; 95% CI, 0.13-0.62; *P* = .003). To be more comparable with results from the Swedish register, age was calculated for patients with different seizure lengths (Table).

**Figure.**
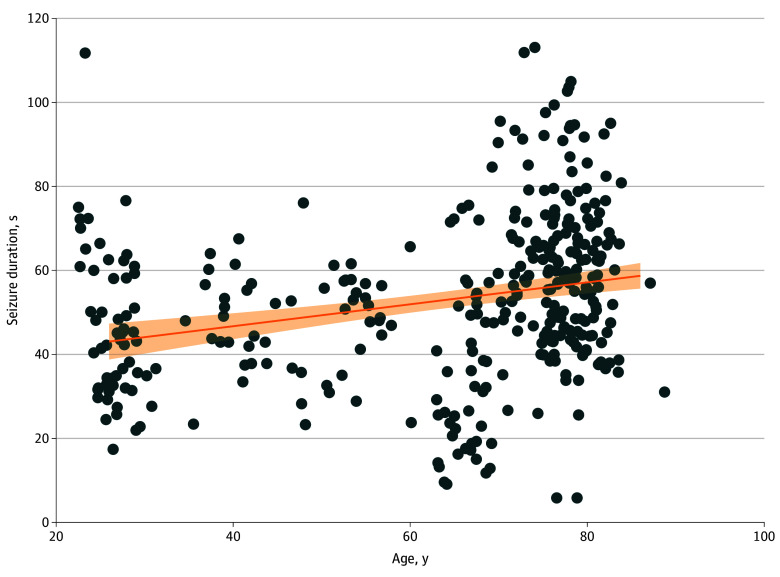
Seizure Duration of the First Electroconvulsive Therapy Session vs Age Each dot represents a single patient. A linear fit (solid line) with 95% CI (shaded area) is shown.

**Table.** Seizure Duration and Age at First Electroconvulsive Therapy Session

Seizure duration, s	Patients, No.	Mean age (SD) [range], y
<30	18	64.9 (10.1) [27-78]
≥30 s and <40	34	50.1 (19.9) [28-86]
≥40 s and <50	59	55.4 (21.4) [26-82]
≥50 s and <60	87	62.6 (19.6) [26-84]
≥60 s and <70	74	66.3 (19.0) [26-84]
>70	79	72.1 (14.4) [26-82]

## Discussion

To our knowledge, we report for the first time an association between age and seizure duration at the first ECT session. This result resolves the paradox of the Swedish registry study that longer seizures at the first ECT are associated with a better response to ECT. Since, according to our data, longer seizures are associated with older age and older age is known to correlate with better response to ECT,^4^ the apparent contradiction is reconciled. However, since these new data seem to fly in the face of the accepted clinical wisdom that young patients have longer seizures at the first treatment, and because seizure duration can be influenced by a range of extraneous factors, we await replication or contradiction by other ECT researchers.

Results regarding seizure duration are always complex and should not be overinterpreted or directly translated into clinical decisions, since observational group-level data of a dynamic treatment effect may have limited ecological validity at the individual patient level. We also know that very long seizures may have unfavorable consequences. Adverse cognitive effects, delirium, and the potential for status epilepticus are concerns with very long seizures.

Of course, seizure duration is not the only marker of seizure adequacy. Various EEG and neurophysiological markers of seizure potency have been proposed and associated with clinical outcomes,^5^ thus the adequacy of a therapeutic seizure may be described in multiple ways.^6^

ECT remains the reference standard in the treatment of major depression. However, the identification of clinical or treatment characteristics that may be associated with treatment success still merits considerable attention.

## Notes

Supplement.
Data Sharing Statement
