# Supplementary material for: Age and First Seizure Length in Electroconvulsive Therapy
Source: JAMA Netw Open. 2025 May 22;8(5):e2512092. doi: 10.1001/jamanetworkopen.2025.12092 (PMC12100448; doi:10.1001/jamanetworkopen.2025.12092)
Supplement: Supplement. — Data Sharing Statement [file jamanetwopen-e2512092-s001.pdf]

Data Sharing Statement

Sartorius. Age and First Seizure Length in Electroconvulsive Therapy. *JAMA Netw Open*.
Published May 22, 2025. doi:10.1001/jamanetworkopen.2025.12092

Data
Data available: No
